# Understanding and Modeling Teams As Dynamical Systems

**DOI:** 10.3389/fpsyg.2017.01053

**Published:** 2017-07-11

**Authors:** Jamie C. Gorman, Terri A. Dunbar, David Grimm, Christina L. Gipson

**Affiliations:** Systems Psychology Laboratory, School of Psychology, Georgia Institute of Technology, Atlanta GA, United States

**Keywords:** teams, team cognition, interpersonal coordination, non-linear dynamics, communication analysis, teamwork

## Abstract

By its very nature, much of teamwork is distributed across, and not stored within, interdependent people working toward a common goal. In this light, we advocate a systems perspective on teamwork that is based on general coordination principles that are not limited to cognitive, motor, and physiological levels of explanation within the individual. In this article, we present a framework for understanding and modeling teams as dynamical systems and review our empirical findings on teams as dynamical systems. We proceed by (a) considering the question of why study teams as dynamical systems, (b) considering the meaning of dynamical systems concepts (attractors; perturbation; synchronization; fractals) in the context of teams, (c) describe empirical studies of team coordination dynamics at the perceptual-motor, cognitive-behavioral, and cognitive-neurophysiological levels of analysis, and (d) consider the theoretical and practical implications of this approach, including new kinds of explanations of human performance and real-time analysis and performance modeling. Throughout our discussion of the topics we consider how to describe teamwork using equations and/or modeling techniques that describe the dynamics. Finally, we consider what dynamical equations and models do and do not tell us about human performance in teams and suggest future research directions in this area.

## Why Study Teams As Dynamical Systems?

A team consists of two or more people that work interdependently toward a common goal (Salas et al., 1992). Counter to many approaches in psychology, understanding teams involves not just understanding isolated mental and behavioral processes in the individual but demands theories and models for how interacting with other people shape thought and behavior in real time. We argue that many approaches aimed at studying interpersonal dynamics, such as social psychology, tend to locate explanations of psychological phenomena within the individual, rather than actual interactions, which is a shift that team psychology demands (Cooke et al., 2013). Because so much of the human condition is based on interacting with other people, we argue that a shift toward interaction- and systems-based psychology, which working with teams entails, touches on a foundational issue in psychological science. For example, a central question when working with teams is, “How do real-time interpersonal processes change the way a person thinks and behaves?” In this article, we advocate a dynamical systems approach for answering this type of question. In this light, teams are viewed as a system of coupled elements that interact over time to produce patterns that are themselves not contained within the team’s members. In order to present a framework for understanding teams as dynamical systems, we first examine the concept of a system and what it means for team psychology.

To appreciate what a dynamical system is, we should first examine the concept of a system (Turvey, 2009). Whereas a system exists independently of whether or not it is recognized as a system (i.e., when something is part of a system, it behaves differently than if it were not a part of that system), *systems thinking* is a matter of perspective. For an astronomer, for example, we suppose the galaxy is a system, and the earth is an element of the system; for a climatologist, the earth is a system, and the earth’s atmosphere is an element of the system etc. In other words, systems (and subsystems) can have fuzzy boundaries, but the important point is that when we use the word “system,” we invoke explanations and understanding precisely at the system-level, rather than the constituent elements of the system (Chapanis, 1996). For example, by focusing on individual-level properties that exist outside of the team in action, “aggregate” views of team cognition that focus on alignment and complementarity of team member knowledge (see DeChurch and Mesmer-Magnus, 2010, for a discussion) present a non-system explanation of team cognition, whereas by focusing on interactions, more “holistic” approaches that view team cognition as the cognition that happens *while* team members interact (Cooke et al., 2013) present more of a systems explanation of team cognition.

At its most basic, the concept of a *dynamical* system (Abraham and Shaw, 1992) simply introduces a temporal element for understanding system behavior. In psychological terms, “dynamical” denotes an emphasis on process (in addition to structure) in understanding and modeling psychological phenomena (Thelen and Smith, 1994). The emphasis on process is important, because when elements are dynamically linked in a system, the ways in which those elements act are different than when those links are absent (Morgan, 2010). Put differently, behaviors can *emerge* at the system level that are not encoded at the level of isolated elements. This concept is captured in Kozlowski and Klein’s (2000) distinction between compositional and compilational emergence in team cognition. Compositional emergence means that properties at the team level (e.g., team knowledge) are isomorphic to properties at the individual level (e.g., sum of individual knowledge). Compilational emergence means that properties at the team level are non-isomorphic to properties at the individual level, where team properties only emerge through the process of team interaction (DeChurch and Mesmer-Magnus, 2010). We take the latter compilational form of emergence as a more general view of how teams work, wherein team interactions dynamically shape team members’ thoughts and behaviors in ways that cannot be known *a priori*.

The fundamental psychological question we started with was how interpersonal processes shape human thought and behavior. Teams are ideal for addressing this question, and dynamical systems provide a powerful theoretical framework for understanding how mental and behavioral processes in the individual are shaped through teamwork. We study teams as dynamical systems because it allows us to directly address the question of how the system shapes element behavior in order to make predictions about future states of the system and the elements in it. By the end of this article, we hope to demonstrate three general principles based on this approach:

(1) Local variability ensures global stability, and global stability entails local variability: Although team interactions can be highly variable and unpredictable on small (“local”) timescales, they are necessarily so in order to maintain stability and predictability of the team on larger (“global”) timescales.
(2) From heart rate variability (Peng et al., 1995) to postural control (Collins and De Luca, 1995), local variability with global stability is a principle that characterizes processes operating at different levels of analysis. Similarly, local-global dynamics in teams are substrate-independent and occur across perceptual-motor, cognitive-behavioral, and neural levels of analysis.
(3) Extending Principles 1 and 2, “cross-level” effects occur between levels of analysis, such that we can gain insight into dynamic processes on one level of analysis (e.g., cognitive-behavioral) by engaging and/or observing the dynamics at another level of analysis (e.g., neural).

We begin by explicating several concepts that will aid in understanding how a dynamical systems approach has been applied to teams.

## Dynamical Systems Concepts in the Context of Teams

Having introduced the general notion of dynamical systems, in this section we describe several concepts of dynamical systems that we have found useful for the study of teams. We describe attractors, perturbation, synchronization, and fractal (power-law) concepts and how they relate to the study of teams.

### Attractors

An attractor is a behavior that a system settles on over time after (possibly) displaying initial transient (settling-in) behavior (Abraham and Shaw, 1992). In predicting system behavior, the system will gravitate toward the attractor, regardless of where it “starts out at” or is “pushed to” by an outside force (e.g., a perturbation; see below). Some attractors are inherently *stable*, such that if the system is pushed away from the attractor it quickly returns to the attractor. Some attractors are *unstable*, such that if the system is pushed away from the attractor, it will be hard to return to the attractor. Other attractors are *metastable*, such that stability must be maintained through active control (a teamwork example is provided later). Sometimes the attractor is cyclical and forms oscillations. For example, pendulum clocks have an oscillatory attractor. In teams, attractors and their stability have been researched in motor coordination and communication processes (described later), where the formation of behavioral attractors for adapting to changing environmental demands has been a central issue (Gorman et al., 2010a,b; Gorman and Crites, 2015).

### Perturbation

A perturbation is an outside disturbance to a system that forces either a reorganization of the behavioral trajectory toward an attractor or moves the system toward a new attractor (Abraham and Shaw, 1992). The effect of a perturbation on the system depends on the system’s stability. A perturbation to a highly stable system is unlikely to shift the system’s behavior to a new attractor. Conversely, a system that is attempting to reach an attractor state during the initial transient period will be highly impacted by a perturbation because system behavior is not stably tied to an attractor. In this respect, the system’s response to a perturbation can be used either as an index of attractor stability (its “relaxation time”) or to “push” the system around its coordination space in order to influence attractor development (Schöllhorn et al., 2006; Frank et al., 2007; Gorman et al., 2010b). In teams, perturbations and stability have been researched in the context of team longevity and training to develop adaptive teams that respond effectively to novel task demands and events in the environment (Gorman et al., 2010a,b).

### Synchronization

Synchronization is a phenomenon where two or more coupled oscillatory processes become coordinated in time across some proportion of frequency (e.g., 1:1, 2:1; Strogatz, 2004). *Coupling* simply means that the processes have some form of interaction with each other. For example, if two pendulum clocks having oscillatory attractors are coupled by placing them on the same surface, the pendulums couple through the surface and eventually oscillate together in time (i.e., synchronize). The synchronization that is observed over time is a new attractor that may not correspond to the natural frequencies of the uncoupled oscillators. Synchronization is an important concept for teams because it describes the impact team members have on each other when they are *informationally* coupled (e.g., through perceptual channels; through communication). Moreover, there are different types of synchronization that can occur (e.g., different frequency proportions, 1:1; 3:2; 7:5; etc.) between team-member inputs. Synchronization can occur during interpersonal coordination both unintentionally and intentionally (Richardson et al., 2005, 2007; Varlet and Richardson, 2015). In teams, synchronization has been researched in communication and team neurophysiology (Stevens and Galloway, 2014, 2016; Gorman et al., 2016), physiological synchronization (Guastello, 2016; Guastello et al., 2016), and in perceptual-motor synchronization (Gorman et al., 2017).

### Fractals and Power Laws

Fractals (Mandelbrot, 1967) model either spatial or temporal processes in which similar patterns occur across multiple scales (e.g., timescales) of measurement. To say that a system exhibits temporal fractal structure, for example, means that it displays a temporal nesting property such that smaller copies of a pattern are nested within larger copies of the pattern, a property called scale-invariance. Scale-invariant processes are fit by a power-law distribution (Schroeder, 2009). Power laws are a signature of self-organization (Bak, 1996) and long-memory effects (Beran, 1994). Self-organization is a process wherein order at the global scale emerges from and constrains component behavior at the local scale (Kelso, 1995), and long-memory effects are correlations that persist over longer timescales than those that characterize local variability within the system (Beran, 1994). When those correlations are positive, it is called *persistence*, and when they are negative, it is called *antipersistence*. It should be noted that system behavior can self-organize around other attractor states (e.g., fixed point; oscillatory); however, we will focus on how teams self-organize around metastable and critical states that exhibit fractal and long-memory dynamics. In psychology, power laws capture fractal scaling in cognitive processes (Gilden et al., 1995; Van Orden et al., 2003) and learning curves across groups of learners (Newell et al., 2001). Fractal scaling has been observed in interpersonal tasks when people match complex movement and communication patterns (complexity matching) that vary across local and global scales (Marmelat and Deligniéres, 2012; Abney et al., 2014; Fine et al., 2015; Coey et al., 2016). In teams, power laws have also been researched in the formation of long-memory in team communication (Gorman, 2005) and in team perceptual-motor learning (Gorman and Crites, 2015), whose timescales extend beyond the memory limitations of the individual. In accordance with Principle 1, fractals and power laws distill what is lawful at the global scale from what appears to be “messy” or “noisy” at the local scale.

## Team Dynamics Across Levels of Analysis

Just as there are different *scales* of analysis (i.e., local vs. global; short timescale vs. long timescale), there are also different *levels* of analysis, including perceptual-motor, cognitive-behavioral, and neural. From a systems perspective, just as processes are temporally linked across scales of analysis, they are physically and informationally coupled across levels of analysis. Therefore, a challenge from the systems perspective is to learn how team dynamics are reflected across different levels of analysis. For example, how are more overt processes observed at the perceptual-motor and cognitive-behavioral levels (e.g., action; communication) coupled with more covert physiological processes at the neural level? In the remainder of this section we present research that examines the unifying dynamical principles outlined above (Principles 1–3) across perceptual-motor interpersonal dynamics, cognitive-behavioral communication patterns in teams, and neural synchronization as a function of team communication patterns (“cross-level” effects). In these sections we also present unifying concepts that get at the question of how team processes shape team members’ thoughts and actions in the form of unintentional synchronization, self-organization, and long-memory effects.

### Team Dynamics at the Level of Perceptual-Motor Coupling

This section describes research on interpersonal synchronization, where behavioral attractors for interpersonal coordination include 1:1 synchronization and more complex (e.g., 3:1) forms of synchronization. The results described in this section begin to demonstrate how team dynamics structure individual behavior. Moreover, in this section we begin to illustrate how the general dynamical principle that teams perform more variable patterns on local scales that contribute to coherence and consistency on a global scale (Principle 1) is realized at the perceptual-motor level of analysis.

**FIGURE 1:**
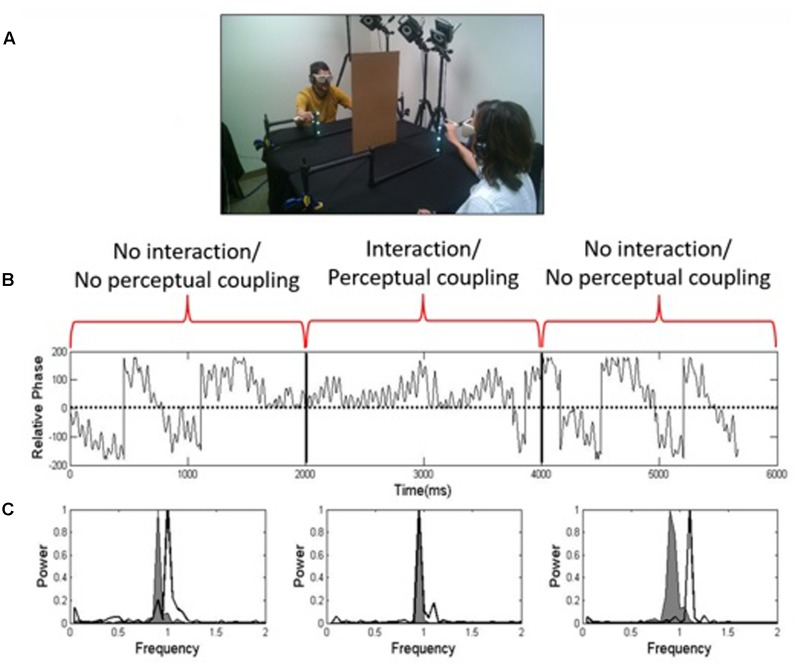
**(A)** A task demonstrating how perceptual coupling and interpersonal interaction induces spontaneous synchronization between people; **(B)** relative phase of participants’ finger oscillations over a one-minute trial; **(C)** power spectra indicating the peak frequencies of participants’ finger movements when vision is occluded (left), un-occluded (middle), and once again occluded (right) (from Gipson et al., 2016; reprinted with permission).

One demonstration of perceptual-motor coupling is based on an interpersonal synchronization phenomenon reported in a large number of studies (e.g., Schmidt et al., 1990; Amazeen et al., 1995; Richardson et al., 2007; Ouiller et al., 2008; Gipson et al., 2016). In one version (Ouiller et al., 2008; Gipson et al., 2016) the demonstration involves two people sitting and facing each other while performing oscillatory finger movements (i.e., oscillating the index finger up and down in the vertical direction; **Figure 1A**). From these finger oscillations, we measure the relative phase (Kelso, 1995; the difference in the phase angles of each person’s finger oscillations; **Figure 1B**) and peak frequencies of their movements (**Figure 1C**). Critically, they cannot always see each other. Visual coupling (being able to see each other’s movements) is used to induce the spontaneous interpersonal dynamics effect. As shown in **Figure 1A**, visual coupling is controlled using visual occlusion goggles. Participants’ instructions are to oscillate their right index finger at a comfortable pace when they hear a start beep. For the first third of the trial, the goggles are occluded (no visual coupling). Notice in the power spectrum in **Figure 1C** the gray and white curves have different peak frequencies during the first third of the trial, which corresponds to the comfortable oscillation speed of each participant with goggles occluded. The only other instruction participants receive is “when you can see, look at the other person.” During the second third of the trial, the goggles are un-occluded, and they can see each other. This visual coupling is accompanied by spontaneous 1:1 synchronization, represented by a shift in relative phase toward zero (**Figure 1B**) and a spontaneous overlap in their peak frequencies (**Figure 1C**) during the middle third of the trial. That is, with no guidance, dyads unintentionally drift toward a state of 1:1 synchronization, the natural attractor of the system. What is revealing is that it is not at a movement frequency that either participant naturally prefers; it is a new behavior that emerges out of interpersonal interaction. Related to the question we started with in the section “Why Study Teams as Dynamical Systems?” this is an example of how interpersonal interaction can change a person’s behavior in unexpected ways. The last third of the trial shows how participants’ movements drift apart when the goggles are once again occluded (no visual coupling). However, we have found that the drift is not instantaneous; there is a “social memory” effect (Ouiller et al., 2008; Gipson et al., 2016). That is, when the goggles are once again occluded, there is a carryover of the interpersonal dynamic to subsequent participant behavior.

This phenomenon might be related to mirroring or mimicry (Chartrand and Bargh, 1999). Mirroring is a phenomenon where if you are sitting across from someone and that person folds their arms, then this “activates” something in you, and you unconsciously fold your arms. Mirroring has been argued to be a pervasive phenomenon that is fundamental to all human interaction (Ramachandran, 2000; Rizzolatti et al., 2001). However, we will argue that 1:1 mirroring is but one of an infinite set of interpersonal ratios whose performance can be better predicted by dynamical systems, and from a team psychology standpoint, mirroring may actually be maladaptive. In team settings that require people to coordinate different but contemporaneous behaviors, spontaneous 1:1 synchronization—mirroring—is a tendency that must be overcome. This includes tasks requiring team coordination across more than one set of hands (e.g., robotic and laparoscopic assisted surgery; Bermas et al., 2004; Zheng et al., 2007; Guru et al., 2012; Liu et al., 2014).

**FIGURE 2:**
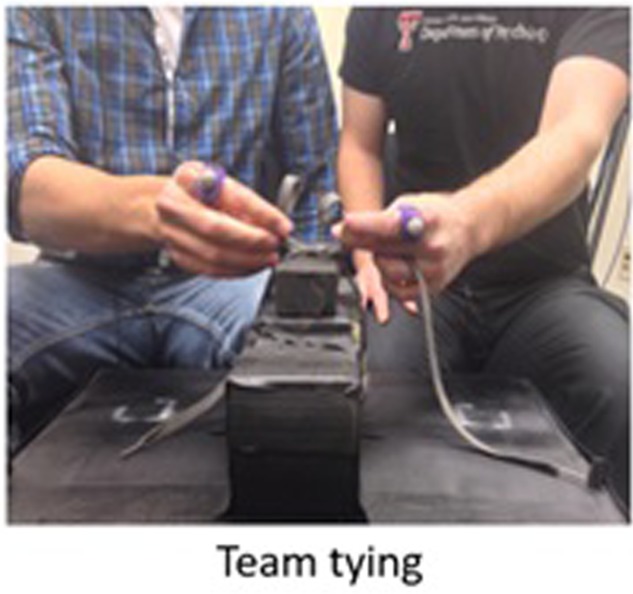
In the team tying task each person handles one lace using one hand but otherwise attempts to tie a shoelace as they normally would.

Gorman and Crites (2015) described how mirroring might negatively impact performance in highly skilled tasks such as surgical knot-tying. The experiment did not use surgeons experienced at knot-tying but participants who were highly skilled in terms of tying their shoes; hence, shoe-tying was a model task for the surgical domain (**Figure 2**). When participants tied individually, their performance curves (trial times for tying a secure knot) were flat, indicating no room for improvement. In terms of individual knot-tying performance, they were experts, limited only by the biomechanical constraints of the task. However, when these experts were asked to work together as a team to tie the knot, there was still a lot to be learned, and their performance demonstrated a learning curve that approached individual performance only after 20 trials. Calculating a measure of between-hand synchronization, the authors found that skilled individual tying is characterized by contemporaneous but independent movements resulting in less synchronization between the hands compared to team tying, and amount of synchronization was positively correlated with trial time (i.e., more synchronization was linked to poorer performance). The authors concluded that when tying as a team, the spontaneous mirroring tendency takes over, and the hands spontaneously synchronize, and participants’ hands are no longer able to move independently, which is what teams apparently need to learn to perform the task effectively. As demonstrated earlier with visually coupled dyads (**Figure 1**), 1:1 synchronization is the natural attractor of the system, which is why non-1:1 synchronization may be so difficult to achieve in a novel team context. We think that the interpersonal skill needed for the novel team tying task may be similar to the skill individuals acquire when learning to play a piano or guitar, where an early challenge is to get their hands to move contemporaneously but independently to produce the desired musical notes (Furuya and Kinoshita, 2008; Furuya and Soechting, 2012).

**FIGURE 3:**
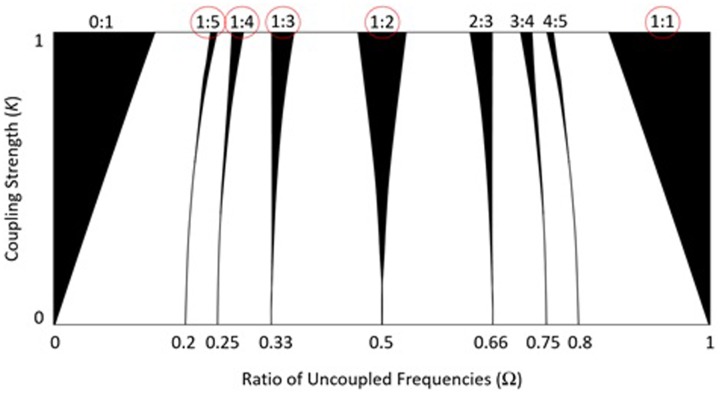
The black Arnold tongues represent the periodic behavior of coupled oscillators in an iterated circle map (𝜃_n+1_ = 𝜃_n_ + Ω - K/2π × sin[2π𝜃_n_];𝜃 = phase of oscillation). The width of the Arnold tongues corresponds to predicted stability of frequency ratios as a function of the intended ratio (Ω) and coupling strength (*K*) between coupled oscillators (performance of the circled ratios is described in the text) (from Gorman et al., 2017; reprinted with permission).

Mirroring is thought to be a pervasive interpersonal dynamic, perhaps rooted in our nervous system (Rizzolatti et al., 2001), but many tasks, such as dancing, playing sports, and coordinating manual labor require that people *not* mirror. Because interpersonal activities are coordinated across and not just within physiological and motor systems, models that are not limited to within-person explanations (e.g., mirroring) are needed. Frequency-locking dynamics provides a model that describes the stability of not just 1:1 mirroring but an infinite range of frequency ratios (e.g., 3:2, which is a more complex, non-mirroring pattern). A graphical depiction of the model for coupled oscillators (e.g., coordinating interpersonal finger oscillations), called the Arnold tongues, is shown in **Figure 3** (Treffner and Turvey, 1993; Peper et al., 1995). For every ratio on the horizontal axis, there is a black Arnold tongue, whose width indicates the stability of the attractor for that ratio. There are an infinite number of Arnold tongues in the interval [0, 1] (i.e., for any ratio), but most ratios are too unstable for people to perform—the skinnier the tongue, the harder it is to keep the ratio. Moving vertically up and down any tongue, it gets wider or narrower, which is a function of the coupling strength between oscillators. Coupling strength can be operationalized as amount of perceptual (e.g., visual; auditory) information exchange between people. Hence the model predicts that while mirroring (1:1 synchronization) is most stable, performance of some non-mirroring patterns (e.g., 2:1) will be more accurate and stable than others (e.g., 4:1) and that increases in coupling strength make the performance of any ratio more accurate and stable.

**FIGURE 4:**
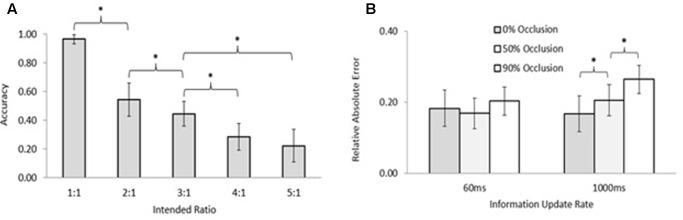
**(A)** Accuracy of interpersonal coordination of mirroring (1:1) and non-mirroring (2:1–5:1) patterns aligns with Arnold tongue predictions; **(B)** visual occlusion (lower coupling strength) makes any ratio less stable (more error) above the critical fusion rate (1,000 ms update rate); however, humans tend to fill in missing information for any ratio when the presentation rate is below the critical fusion rate (60 ms) (from Gorman et al., 2017; reprinted with permission).

Our results using the interpersonal finger oscillation task (e.g., **Figure 1A**) align with these model predictions, but with interesting twists based on inherent properties of the human visual system (Gorman et al., 2017). **Figure 4A** shows accuracy of five simple ratios, one of which (1:1) corresponds to perfect 1:1 mirroring. As the intended ratio moves farther from perfect mirroring, corresponding to narrower tongue widths, performance becomes less accurate. This is not surprising: the more different the movements, the harder they are for people to keep. However, more support for model predictions can be seen in **Figure 4B**, which shows the effect of % visual occlusion (coupling strength) on the stability of any ratio (more error implies less stability). As shown on the right side of **Figure 4B** (1,000 ms), in accordance with model predictions the higher the visual coupling the more stable any ratio. However, it is important to note how the properties of the human visual system can modify these dynamics (the need to account for individual-level properties in the context of team dynamics is addressed in the later section Criticism of the Dynamical Systems Approach and Future Directions). The 60 ms rate in **Figure 4B** is below the critical visual fusion rate (Card et al., 1983), which corresponds to the principle behind motion pictures that if discrete images are put together fast enough, then people will perceive them as a continuous visual stream (Hochberg, 1986). If people are provided with deprived or noisy information *under* the critical fusion rate (e.g., the 60 ms rate), then they tend to fill in the missing coordinative information to preserve interpersonal performance even for more complex, non-mirroring patterns. Based on this, mirroring alone may not explain interpersonal coordination as well as previously thought, or why our perceptual systems fill in more complex, non-mirroring patterns when we coordinate with each other. Systems-level explanations, such as frequency locking, provide additional insight into how people coordinate not only mirroring but also non-mirroring behaviors with each other.

An example of actual team performance that aligns with what we observe in the laboratory can be found in the sport of Double Dutch (Gorman et al., 2017). Double Dutch is a team sport involving two people on either end of two long jump ropes who simultaneously twirl both ropes while another person jumps over the twirling ropes. Working with the National Double Dutch League, we have investigated non-mirroring coordination patterns between rope turners’ and jumper’s movements under the predictions of frequency-locking.

**FIGURE 5:**
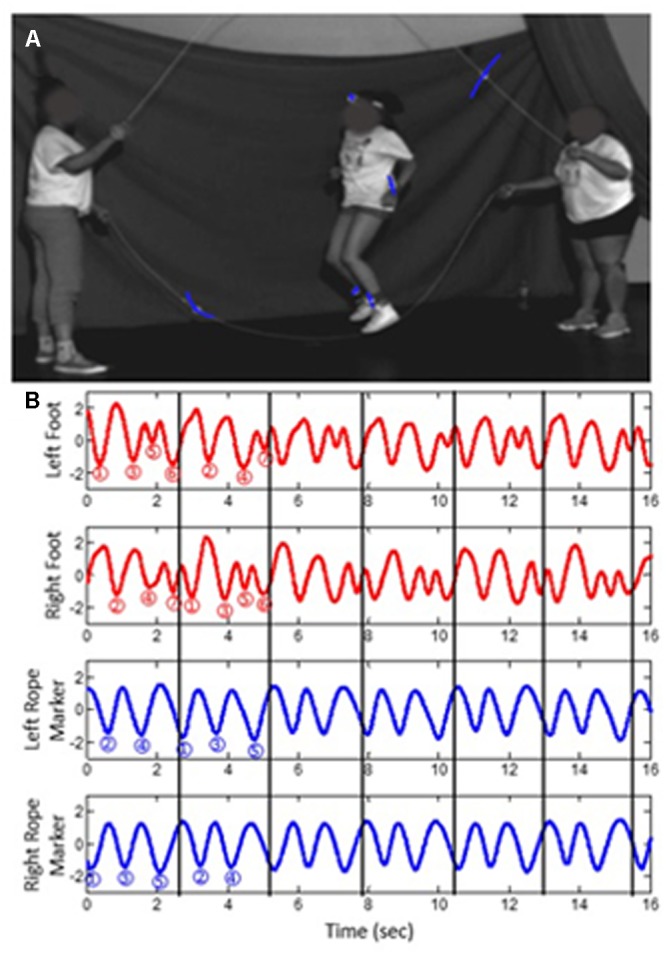
**(A)** A highly skilled Double Dutch team at the National Double Dutch League summer camp; **(B)** performance of a 7:5 (foot:rope) ratio by the team (from Gorman et al., 2017; reprinted with permission).

**Figure 5A** shows a highly skilled team performing a 7:5 footfall-to-rope-turn ratio. Their performance is incredibly consistent (**Figure 5B**), given the predicted difficulty of the ratio. Compared to a 1:1 ratio (mirroring), which even beginners can perform, as they move further from mirroring, they increase their coupling strength through increased visual attention and through rhythmic counting, which is a more cognitive form of coupling. In terms of the model, by increasing coupling strength, they effectively widen any tongue, which allows them to stabilize any ratio.

**FIGURE 6:**
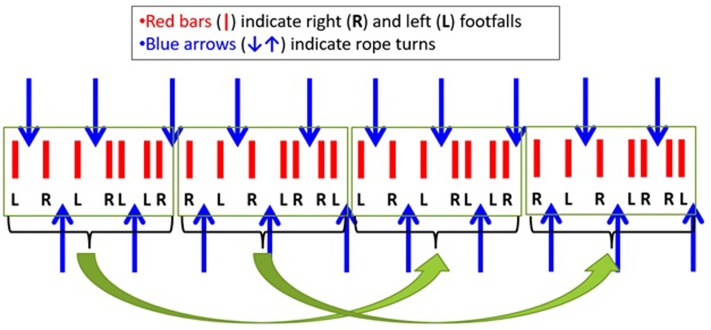
To maintain a stable ratio on a global (overall pattern) scale, teams can vary their patterns on a local (cycle-by-cycle) scale.

When performing this complicated pattern, participants modify the 7:5 pattern cycle-by-cycle. That is, for one 7:5 grouping of movements, they perform a particular pattern, and for the next 7:5 grouping of movements, they perform a different pattern, such that the pattern is locally variable but globally stable. As shown in **Figure 6**, the way the red footfalls are interspersed with the blue rope turns varies on a local (cycle-by-cycle) scale but is stable on a global (overall pattern) scale. This recounts the idea that teams perform more variable patterns on local scales that contribute to coherence and consistency on a global scale (Principle 1), which, as discussed next, appears to be something that is fundamental to team performance across levels of analysis (Principle 2).

### Team Dynamics at the Level of Cognitive-Behavioral Coupling

This section extends Principle 1 to the cognitive-behavioral level of analysis, demonstrating how local-global dynamics occur across different levels of analysis in teams (Principle 2). We focus on how individual communication and coordination behaviors are dynamically structured to maintain team effectiveness at the global scale. Moreover, we demonstrate how team dynamics at the cognitive-behavioral level compel team members to communicate in somewhat unpredictable ways at a local scale that nevertheless contribute to coherence and consistency—here, fractal and power law dynamics—on a global scale.

**FIGURE 7:**
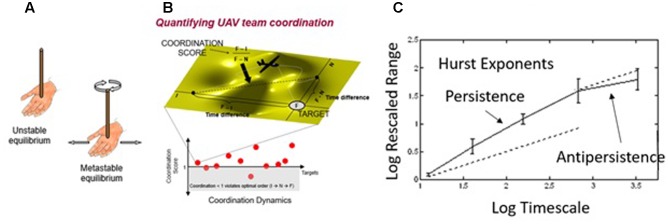
**(A)** The inverted pendulum; **(B)** UAV team coordination score; **(C)** short-range persistence and long-range antipersistence of the coordination score follows inverted pendulum dynamics.

Another useful model for team dynamics is the inverted pendulum. If this is not familiar, think of trying to balance a rod upright in your hand (**Figure 7A**). The challenge is to maintain the upright balance although the rod’s natural tendency—its natural attractor—is to fall to the ground. The rod balanced upright is a metastable state that is created when your hand movements counteract the natural tendency of the rod to fall to the ground (Treffner and Kelso, 1999). The hand movements may appear random or unpredictable, but this behavior is necessary for keeping the overall system (i.e., rod balanced upright) stable and predictable on a global scale. Similarly, although team members share a common goal, because they operate in dynamic environments the natural tendency of team members is to behave in ways that might seem unpredictable on a local scale but necessarily so in order to maintain team effectiveness on a global scale (Gorman et al., 2010a). In this regard, team dynamics contains a metastable state that is maintained through team interaction at the cognitive-behavioral level of analysis (e.g., team communication).

Interactions among three-person uninhabited air vehicle (UAV) teams—a photographer, pilot, and navigator working together to take ground photos—demonstrate these dynamics (Gorman et al., 2010a). We used timestamps of critical team coordination events needed for taking photos of ground targets and combined these into a coordination score (**Figure 7B**). The coordination score captures the temporal relations of the critical coordination events for each ground target and exhibits inverted pendulum dynamics (**Figure 7C**). On short timescales we see persistence, and on longer timescales we see antipersistence. In the inverted pendulum, drifts away from straight up in a particular direction (persistence) occur on short timescales, and these drifts are counteracted by corrections back to straight up (antipersistence) on longer timescales. Similarly in the UAV teams, short timescale (local) variability in terms of a particular target coordination pattern is bounded by a longer timescale (global) coordination pattern across all targets (Gorman et al., 2010a). Again, this is the theme of more variable patterns on local scales that contribute to coherence and consistency on a global scale (Marmelat and Deligniéres, 2012; Principle 1).

**FIGURE 8:**
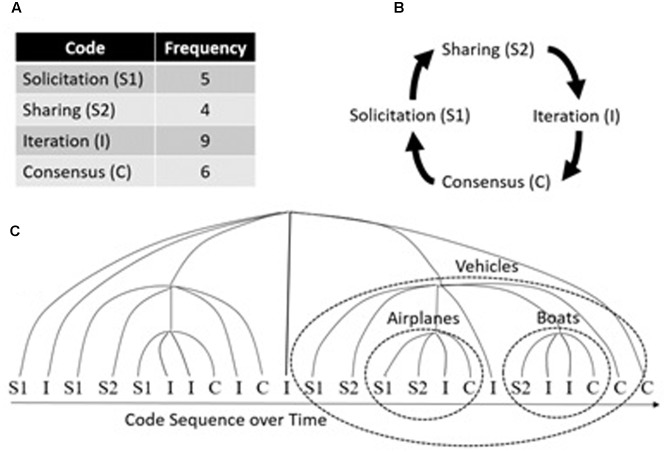
**(A)** Code frequencies for the sample sequence of codes; **(B)** a simple linear transition (Markov) model of the most probable Lag-1 code transitions; **(C)** hypothesized temporal nesting (i.e., fractal structure) of code transitions organized around task-relevant communication.

This principle is also apparent in the temporal nesting of communication behavior over time. **Figure 8** shows a sequence of communication codes obtained from transcribing a team’s conversation, separating it into utterances, and coding them using a mutually exclusive set of communication types, comprised of Solicitation, Sharing, Iteration, and Consensus (Gorman et al., 2009). Looking at the code sequence over time, it appears random, perhaps resembling a memoryless Poisson process. In that case, a Markov model (**Figure 8B**) can account for local variation in the sequence of codes (i.e., which code tends to follow which), as indicated by the smaller ovals in **Figure 8C**. But, there is a good amount of unexplained variation using this approach (Gorman et al., 2009), leading one to wonder how accurately a Markov model describes the process that generated the sequence of codes.

As we incorporate longer timescales, we see that the conversation continues to exhibit the transition structure of **Figure 8B**, but operating on a longer timescale (i.e., the larger oval, “Vehicles,” in **Figure 8C**), suggesting a temporal fractal structure for team communication. For example, on short timescales you might find these code transitions in a discussion of airplanes and boats, but those short timescale conversation transitions are nested within a longer timescale conversation about vehicles in general. Hence, though linear transition models such as Markov models do account for some local variation during conversation, we must also account for non-linear (fractal) nesting of conversation topics across longer timescales. More recently, we have quantified this process in action-based teams who coordinate across real-time perception-action links and decision-making teams who coordinate across more cognitive, planning links (for a discussion of these team types, see DeChurch and Mesmer-Magnus, 2010).

Dunbar and Gorman (2014) examined the impact of task constraints on the temporal fractal structure of team communication. In this study, dyads performed either an action-based task or a decision-making task selected to introduce different team interaction constraints. After teams performed their task, their communication was transcribed and coded using Butner et al.’s (2008) coding scheme into three mutually exclusive code types: Facts (i.e., communication focused on perception and action), Interpretations (i.e., communication focused on cognitive processing), and Conversation Regulation (i.e., communication focused on maintaining the flow of conversation). The temporal distribution of each code was evaluated for each team’s transcript and converted into slopes of the line relating log scale size (possible number of intervening codes between each occurrence of the code [e.g., Fact] being analyzed) by log frequency (frequency count of the number of occurrences of intervening codes at each scale size) to test for a power-law relationship (Brown and Liebovitch, 2010).

The results of this study indicated that communication specific to the type of team task exhibited fractal (power-law) scaling. Specifically, Fact-based communication was more fractal for action-based teams, and Interpretation-based communication was more fractal for decision-making teams. These results confirmed that the temporal nesting (i.e., fractal structure) of code transitions was organized around task-relevant communication. (As expected, Conversation Regulation was similar for both team types and did not exhibit temporal fractal structure).

To determine whether these patterns were generated by a self-organization process, we compared the power-law distribution fits to a memoryless Poisson process. Memoryless Poisson events are only locally variable (waiting time parameter) and follow an exponential distribution. Both Facts and Interpretations were significantly better fit by a power-law rather than an exponential function (there was no difference for Conversation Regulation). We think that the global self-organization of team communication commences when a system (team) is continuously balanced on the verge of change as new information is added (as the conversation evolves) at the local scale (i.e., self-organized criticality; Bak, 1996). Hence, the global order of conversation evolves out of locally variable communication inputs and evolves most clearly for task-relevant communication acts.

Systems characterized by self-organization also exhibit long-memory (Beran, 1994). Long-memory can be thought of as a type of memory that is not contained in individual elements of the system (e.g., working memory) but in the history of interactions among system elements (i.e., system-level memory). In terms of team communication, the presence of long-memory means that team members’ interactions are not just intentional acts at a local scale but are informed by the history of interactions at the global scale. We have observed the development of long-memory effects in medical and military teams in terms of the coherence of their conversation as they communicate over time.

**FIGURE 9:**
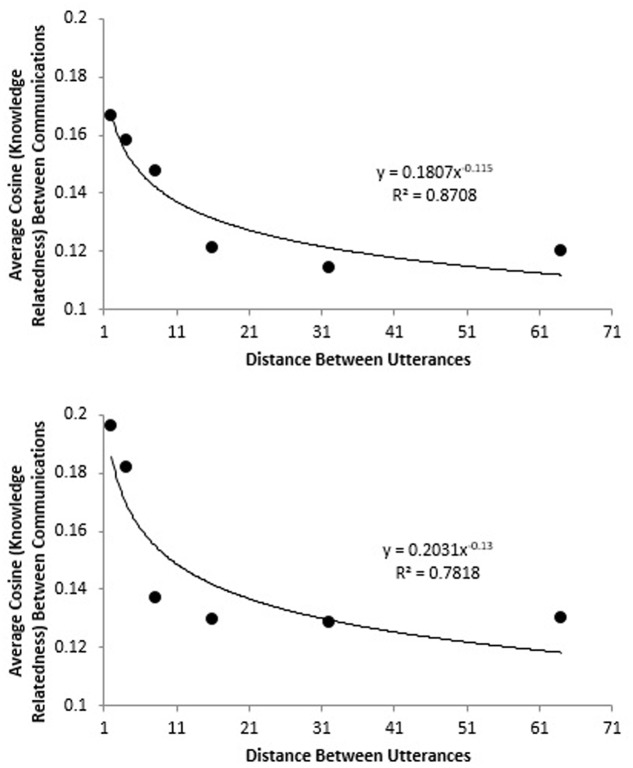
These figures show how the knoweldge relatedness of communication diminishes as timescale (distance between utterances) increases. The communication of the experienced team in the top panel has more long-memory than the communication of the novice team in the bottom panel.

The Latent Semantic Analysis (LSA; Landauer et al., 1998) cosine measures the relatedness (“coherence”) between any two pieces of discourse (e.g., any two utterances; any two transcripts; etc.). The timescale on which the cosine measure demonstrates coherence can be used to assess the characteristic timescale on which teams communicate knowledge, a measure of the long-memory of a team (Gorman, 2005). **Figure 9** shows how cosine (knowledge relatedness) diminishes as the timescale (distance between utterances) is increased for two medical teams (these teams are described in the study by Stevens et al., 2016). The steeper drop off for the team in the bottom panel suggests that their discourse has a shorter timescale of coherence (their conversation has a “shorter memory”); by contrast, the team in the top panel has a longer timescale of coherence (their conversation has a “longer memory”). In this between-team comparison, both teams performed a simulated medical procedure, but the team with shorter memory was a novice team, whereas the team with longer memory had significant experience working together.

Another study by Gorman (2005) used the LSA cosine method to investigate within-team changes in long-memory in UAV teams. Teams learned to take photos of ground targets over five 40-min mission segments. The first four missions were low workload, followed by a high workload mission. The results indicated that the amount of long-memory in team communication increased from Mission 1 to Mission 4. In Mission 1, long-memory had not been established, and communication patterns were only locally variable. However, by Mission 4 long-memory had been established, such that team communication displayed persistence over short-to-medium timescales and anti-persistence over longer timescales. Like the inverted pendulum, there was an interplay between positive and negative feedback on local and global scales that structured team communication, which is a general characteristic of self-organized and long-memory processes. The long-memory effect weakened at Mission 5, however, indicating that the high workload condition may have regressed teams back toward a novel state, similar to Mission 1, before long-memory had been established.

The studies described in this section are consistent with Principle 1, that local variations in intentional communication behaviors are dynamically structured to maintain team effectiveness and coherence at the global scale. Moreover, we would argue that as with unintentional synchronization, global patterns in team communication can compel team members to interact in unexpected ways (Gorman and Cooke, 2011; Gorman, 2014). In combination with the studies described in the section “Team Dynamics at the Level of Perceptual-Motor Coupling,” and in accordance with Principle 2, we see similar patterns of local-global dynamics at work across perceptual-motor and cognitive-behavioral levels of analysis. In the next section, we turn to Principle 3 by examining research on team dynamics across levels of analysis.

### Cross-Level Effects between the Cognitive-Behavioral and Neural Levels of Analysis

In this section, we extend Principle 2 by tying dynamics together across neural and cognitive-behavioral levels of analysis (Principle 3). In particular, we describe our findings on cross-level effects wherein changes in communication patterns are associated with changes in neural patterns and how environmental perturbations simultaneously impact dynamic signals at both levels of analysis.

One way of examining neural processes in the context of team dynamics is by comparing them to simultaneous cognitive-behavioral processing in team cognition, such as team communication. When people communicate, their neural activity often becomes synchronized. This synchronization is present as a spatial and temporal correlation between the speaker and listener’s neural activity (Stephens et al., 2010). This correlation occurs at a delay, often with the listener’s neural activity preceding the speaker’s neural activity (Stephens et al., 2010). It is argued that this neural coupling serves as a method for how brains successfully convey information between interacting individuals. In this context, *cross-level effects* examine how neural coupling, in the context of neural synchronization across team members, is affected by changes in team communication patterns (Gorman, 2014; Gorman et al., 2016).

Gorman et al. (2016) investigated cross-level effects in novice and experienced submarine crews. The communication variable was the LSA vector length, which quantifies the degree to which an utterance relates to the domain of discourse. The neural activity variable was the Shannon entropy (Shannon and Weaver, 1949) over a series of electroencephalography (EEG) neurodynamic symbols that describe the distribution of neural activity across team members. Neurodynamic entropy essentially indicates how much the neurophysiological distribution is changing across team members over time (Stevens and Galloway, 2014, 2016, 2017). The higher the entropy, the more the distribution of neural activity is changing; the lower the entropy, the less the distribution is changing, and the more neurally synchronized the team. Lagged cross-correlations between the LSA vector length of each utterance and mean entropy during each utterance were calculated to determine the presence of cross-level effects. Peak cross-correlations indicated that changes in communication patterns are immediately reflected in changes in neural synchronization for novice crews (i.e., peak cross-correlation at Lag-0) but that changes in neural synchronization tend to be preceded by changes in communication pattern for expert crews (i.e., lead-lag effects). This suggests that as people continue to work as a team, communication can influence neural coupling by dynamically entraining the distribution of neural activity across team members. Hence, team dynamics at the neural and cognitive-behavioral levels of analysis are coupled, and this coupling occurs across a temporal lag as team members continue to work together (Principles 2 and 3).

**FIGURE 10:**
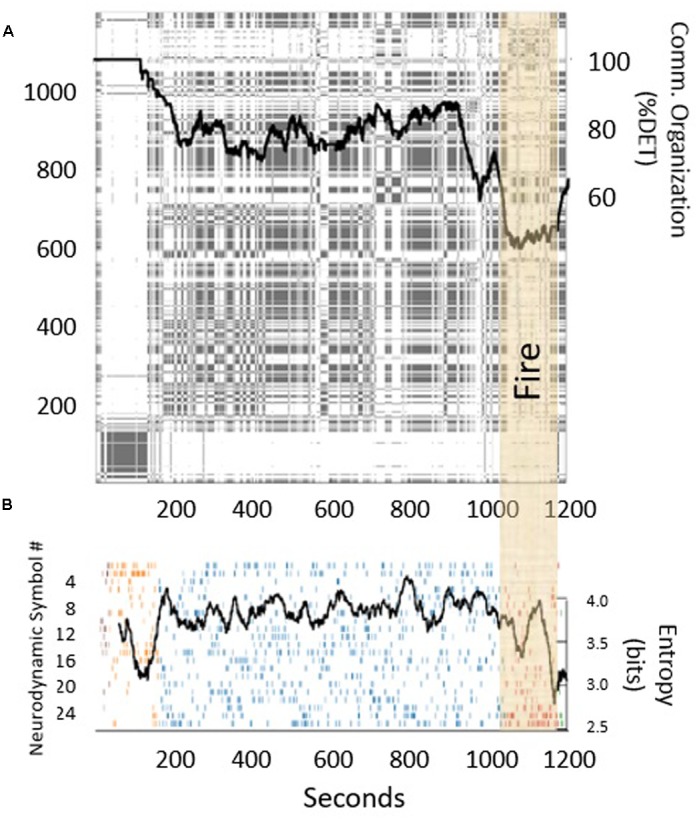
**(A)** Discrete recurrence plot of speaker turn-taking in a medical simulation. The black trace measures the communication determinism (larger values mean more orderly; smaller values mean more random) around the main diagonal using a moving window of size 150. **(B)** The black trace measures the simultaneous neurodynamic entropy across team members.

More evidence of cross-level effects can be seen in research on medical teams. Stevens et al. (2016) monitored EEG signals in surgical teams and measured their neurodynamic entropy while simultaneously capturing their communication activity. **Figure 10A** shows one team’s discrete recurrence plot (discrete RP; Gorman et al., 2012a) of turn-taking during team communication. For a sequence *x* of length *N*, the discrete RP is an *N* ×*N* symmetric matrix, where if the value of *x*(j) is identical to the value of *x*(i), then a dot (“recurrent point”) is plotted at *x*(i,j) in the RP. Note that the main diagonal in the RP is completely filled in because it is the one-to-one plot of the sequence against itself at i = j. Changes in how the dots cluster around the main diagonal indicate changes in communication flow (i.e., patterns of who is talking and when) over time. The amount of organization (i.e., how orderly vs. random) in communication flow can be measured by calculating the determinism (%DET) of the cluster of dots around the main diagonal. %DET is calculated as the number of recurrent points forming diagonals divided by the total number of recurrent points (we refer the reader to Shockley, 2005, for other measures that can be calculated). The black trace overlaying the RP in **Figure 10** is a moving window calculation of %DET around the main diagonal. Note the drop in %DET, or turn-taking organization, at about 1,000 s, which corresponds to a breakdown in communication when a fire broke out in the operating room (OR). As shown in **Figure 10B**, this behavioral breakdown as measured by a drop in %DET was associated with a contemporaneous drop in neural entropy in the team (spikes in entropy of communication codes have also been shown to be sensitive to changes in task dynamics; Wiltshire et al., 2017). Specifically, the communication breakdown precedes a negative spike in neural synchronization, which happens when a team mentally locks up due to environmental perturbations and indicates a re-organization of team neurophysiological state (Stevens and Galloway, 2016). Hence, as communication becomes disorganized, and then reorganized, the team’s neural signals display an accompanying re-organization of system state at the neural level (Principle 3).

Having described in the section “Team Dynamics across Levels of Analysis” a series of results underpinning Principles 1–3, we turn to a discussion of the theoretical implications of the dynamical systems approach for conceptualizing psychological processes and human performance in teams.

## Theoretical Implications of the Dynamical Systems Approach to Teams

First, it should be noted that the dynamical systems approach described in this article has many underpinnings in the history of psychology. These include psychological theories that embrace systems thinking, such as the ecological approach (Gibson, 1966), activity theory (Leont’ev, 1981), coordination dynamics (Kelso, 1995; including interpersonal, Richardson et al., 2005, 2007), distributed cognition (Hutchins, 1996), groups as complex systems (McGrath et al., 2000), interactive team cognition (Cooke et al., 2013), dynamical systems in team sports (Grehaigne et al., 1997; Bourbousson et al., 2010; Vilar et al., 2012; Cuijpers et al., 2015), non-linear dynamics in human factors and ergonomics (Guastello, 2017), and systems thinking in human factors (Chapanis, 1996) and human-computer interaction (Barnard et al., 2000). What is different about the dynamical systems approach to teams, and what does it offer team psychology?

Though there are many different approaches to understanding how systems in action affect human behavior, the dynamical systems approach to teams is primarily rooted in objective team coordination/performance metrics and mathematical representations that explain how interpersonal interaction lawfully relates to individual-level variability. One theoretical implication of this involves the so-called “slaving principle” (Haken, 1983), which is the control of system elements by an “order parameter” that captures global coordinative structure. Demonstrations of this principle can be found in interpersonal coordination research (e.g., Schmidt et al., 1990; Amazeen et al., 1995; Richardson et al., 2005, 2007; Ouiller et al., 2008; Gipson et al., 2016). In the context of the slaving principle, variability in individual behavior must be understood in the context of global coordination parameters (e.g., power laws and long-memory effects) that compel team members to behave in certain ways (Gorman and Cooke, 2011). A related implication involves how the perturbation of a system ripples through the system due to the interconnectedness of system elements. For human behavior, the important point is to understand how perturbing one or a few individuals affects and changes the behavior of other, connected individuals. We have empirically demonstrated this idea in training adaptive command-and-control teams (Gorman et al., 2010b; described later) but, moreover, this idea carries implications for how environmental change (broadly construed) impacts the thoughts and behaviors of people embedded in that environment.

Inheriting from some of our theoretical forerunners is that the dynamical systems approach to teams emphasizes the “psychology of active systems” rather than the “cognitive sandwich” (i.e., stimulus, cognitive processing, response) mode of explanation. The dynamical approach to teams focuses on real-time interactions as the appropriate level of psychological inquiry for understanding how other people and our surroundings structure thought and behavior. This is in contrast with the nostalgic view of psychology that aims to understand psychological processes by studying isolated individuals and only later adding real-time interactions as “context effects” once the solitary processes have been understood (Wertsch, 1991). As a matter of course, the difference in analysis is one of beginning with the system as a whole versus trying to integrate components into a system once the components are understood. The result of this is that explanations and models of human behavior that a dynamical systems approach provides (e.g., attractors; long-memory) are unfamiliar to many psychologists and other students of human behavior, whereas traditional explanations and models (e.g., neurons; representations), although attractive to psychologists, do not contain the necessary information to understand how our thoughts and behaviors are shaped by the dynamic interpersonal interactions in which they are embedded.

As embodied in Principles 2 and 3, there is no preferred level of analysis for investigating team dynamics. The dynamics are present across levels of analysis, and the assumption of theory reduction (e.g., that the psychological must be reducible to the biological) and the accompanying bridge laws are not required. Put differently, there is no “fundamental substance” or “unit of analysis” in team psychology; everything is dynamic process (Thelen and Smith, 1994). This does not preclude observing dynamic process on one level of analysis while ignoring others, but it assumes that behaviors on unobserved levels of analysis are simultaneously being shaped by the same dynamics. Hence, the decision to analyze one level of analysis or even to decide what levels of analysis exist may seem somewhat arbitrary. In our experience, the first decision is based on the research question at hand (e.g., is it about overt behavioral acts, or is it about covert neural processes?) and the second is constrained by the equipment available to measure the dynamics (e.g., motion capture vs. voice recordings vs. EEG).

As with any method of inquiry, the dynamical systems approach carries its own characteristic language and style of argument that constrains the types of explanations it can offer (Quine, 1951). Theoretical ideas emanating from the dynamical systems approach to teams will tend to focus on how behavior changes through interpersonal interaction and how global interaction patterns come to structure individual thought and behavior. Moreover, there is no preferred level of analysis; the choice depends on the research question and careful selection of measurement equipment. This is in contrast to approaches that emphasize psychological processes that *must* be localizable within the individual and *must* be understood in terms of a fundamental substance or unit of analysis (e.g., brain function as ultimate theory reduction).

## Practical Implications for Team Training and Assessment

Traditional approaches to team training including crew resource management (Helmreich et al., 1999) and cross-training (Blickensderfer et al., 1998) emphasize the alignment of team member knowledge, skills, and attitudes (KSAs; Salas et al., 2006) to enhance team performance. These approaches have been successful in enhancing team performance (e.g., Marks et al., 2002). We argue that the dynamical systems approach to team training can further enhance human performance under novel conditions in the post-training environment.

Perturbation training (Gorman et al., 2010b) is a team training approach that draws on the systems proposition that when a coordination pattern is perturbed, all team members (not just those directly affected by the perturbation) must readjust their interaction patterns at a local scale to maintain system stability and team effectiveness at a global scale (Principle 1). Well-placed perturbations (e.g., unexpectedly cutting a communication link) exercise the potential coordination space of a team beyond routine conditions by forcing them to develop new solutions for novel coordination problems. The prediction for team training is that by introducing perturbations during team skill acquisition, we increase the flexibility and adaptability of the team members, thereby enhancing team performance in response to novel and unpracticed task conditions. This training approach has precedence in the transfer of motor and verbal learning to novel situations (Schmidt and Bjork, 1992) and in training individual and team sports (Schöllhorn et al., 2006; Renshaw et al., 2010).

In the Gorman et al. (2010b) study, perturbation training led to superior performance under novel task conditions compared to cross-training and procedural training. Teams in the cross-training condition developed shared knowledge to a greater degree than teams in the other conditions and performed just as well as perturbation-trained teams on tests of routine task performance. Compared to cross-training and perturbation training, procedural training led to the least effective teams under both routine and novel task conditions. However, performance under novel task conditions was enhanced through perturbation training compared to both cross-training and procedural training. We think that flexibility in real-time interaction processes induced by perturbation training, rather than shared knowledge or following scripted procedures, enhances team performance by exercising the real-time dynamics that team members need to experience in order to adapt in the post-training environment.

**FIGURE 11:**
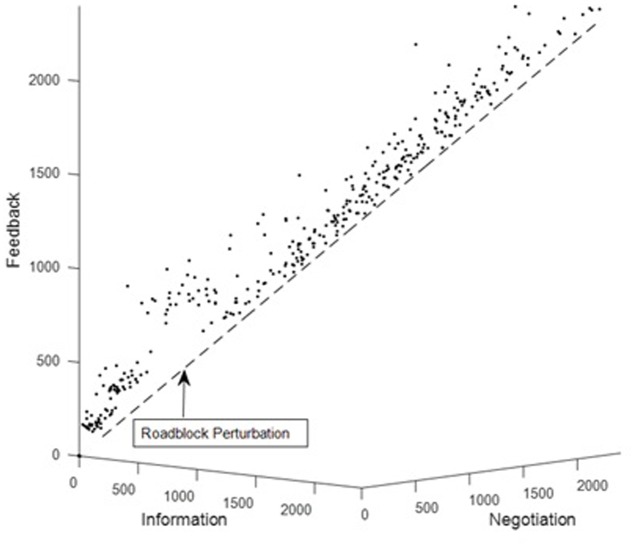
Measuring the team response to a roadblock (“relaxation time”) as a method for team assessment.

Perturbing team coordination is closely related to a systems approach for measuring team situation awareness (team SA; Gorman et al., 2005, 2006; Cooke et al., 2009). This approach involves identifying “roadblocks,” which are novel or unlikely task conditions that require an adaptive and timely coordinated response in order to maintain team effectiveness. In this approach, team SA is assessed as a team’s ability to team overcome roadblocks in a timely manner (Cooke and Gorman, 2009). **Figure 11** shows how the timing of the components of the UAV coordination score from **Figure 7** (the dots) are altered by a roadblock. Under routine task conditions, the dots gravitate toward the diagonal line (the attractor). Roadblock onset occurs at about 500 s, and the dots are “pushed” off the attractor (diagonal line) by the roadblock, corresponding to an alteration of the routine coordination pattern. Two measures of team SA in response to a roadblock are whether the team overcomes the roadblock (i.e., whether the dots gravitate back toward the diagonal line) and the time to overcome the roadblock (i.e., how long it takes for the dots to gravitate back toward the diagonal line). The latter assessment is related to the dynamical concept of *relaxation time*, which is essentially the time it takes for a system to return to its attractor after its trajectory has been perturbed. In actual teams, a roadblock could have catastrophic consequences if a team has a long relaxation time and does not respond appropriately and in a timely manner. For practical purposes, real-time analysis of team coordination can help prevent catastrophic errors caused by delayed team responses.

Team communication, cognition, and coordination give rise to dynamic patterns that change in real time. Breakdowns and unexpected changes in these processes are at least partially responsible for the Challenger Shuttle disaster (Vaughan, 1996), delayed response times to Hurricane Katrina (Leonard and Howitt, 2006), and poor communication in air traffic control in response to the September 11th attacks (Kean and Hamilton, 2004). For team assessment, it is important to detect these breakdowns and roadblocks as they unfold in real-time (Gorman et al., 2012b).

The assumption behind real-time dynamics is that we can meaningfully analyze team interaction data *ad hoc*, as it becomes available, as opposed to *post hoc* (Gorman et al., 2012b). This is plausible due to the “historical” quality of team interaction, such that team communication has long-memory. That is, a current observation in a team communication time series is not independent from previous observations—teams have *momentum* (Den Hartigh et al., 2014)—and this creates temporal dependencies that can be quantified using dynamics (Smith et al., 2008; Gorman et al., 2012b).

**FIGURE 12:**
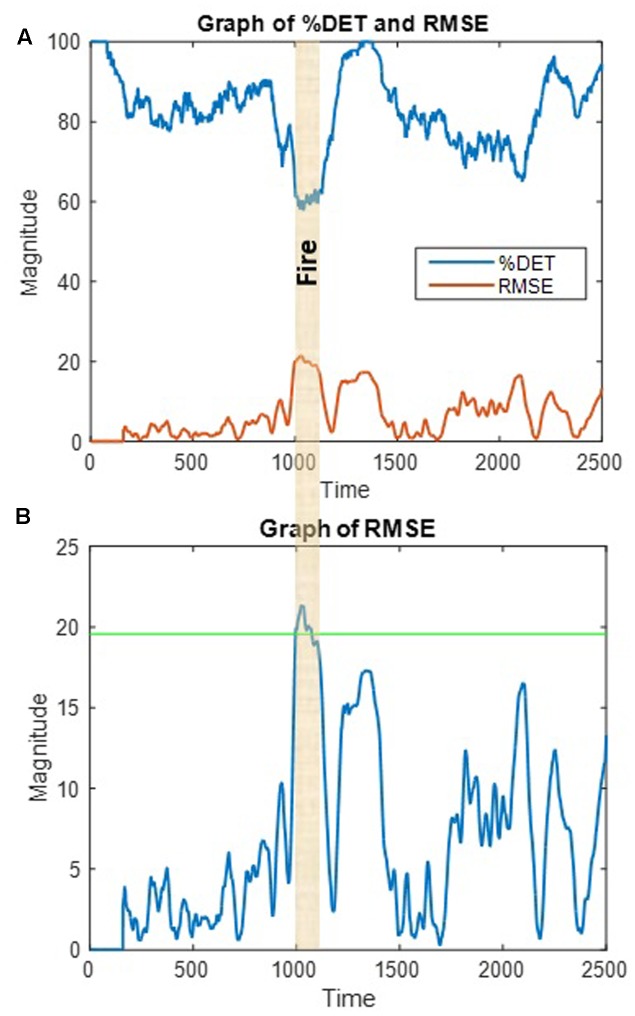
**(A)** Graph of communication determinism (%DET) and root mean square error (RMSE) from a prediction model; **(B)** RMSE relative to a 99% confidence interval (green line) indicates a significant fluctuation in communication pattern (drop in %DET) in response to the fire in the OR.

We have been successful in developing methods to detect teamwork breakdowns and roadblocks in near-real time using turn taking patterns during team communication in different real-time contexts (Gorman et al., 2012b; Grimm et al., in press). Using the non-linear prediction algorithm described by Kantz and Schreiber (2004), we stream in a communication variable and scan it to detect fluctuations in communication patterns that significantly differ from previous observations of the communication variable. The assumption is that as in **Figure 11**, significant fluctuations in team communication patterns correspond to significant environmental perturbations that require a timely response. To illustrate, **Figure 12A** reproduces the determinism time series from **Figure 10A** (top trace) from the surgical team study along with the root mean square error from the non-linear prediction algorithm (bottom trace). The root mean square error is also plotted in **Figure 12B** relative to a 99% confidence interval, which indicates that the fire in the OR corresponded to a significant perturbation to the team’s communication dynamics. Once a significant perturbation is detected, if the team is responding adaptively, then we expect the prediction error to return to a non-significant level in a timely fashion. If not, then some form of outside intervention might be required to effectively address the situation. If the team does not respond at all to a significant environmental perturbation (such as a fire in the OR), then this could reflect a deeper operational issue in need of remedial training.

Real-time analysis is useful for detecting change in dynamical systems in response to a significant environmental perturbation. Applications of real-time analysis can potentially identify significant and harmful changes in the team environment to ensure they are acted on in an appropriate and timely manner. The above illustration described a method of real-time analysis as applied to team communication. However, there is potential for these methods to be applied to perceptual-motor and neural levels of analysis such as those described in other sections of this article (i.e., application of Principles 2 and 3).

## Criticism of the Dynamical Systems Approach and Future Directions

Dynamical systems approaches in psychology have been cautioned to avoid the mistake of drawing generalizations about psychological processes simply because they carry a particular dynamical signature (Rosenbaum, 1998). This is followed by the more general criticism that there is no psychological “mechanism” responsible for producing the dynamics (see Van Orden et al., 2003 for a discussion). Here, mechanism means something like a neural pathway or information-processing component (e.g., working memory) within the individual. Hence, one issue with the dynamical systems approach is that it does not naturally align with the mechanism-within-the-individual explanation so often sought in psychology. Because it is all about process and interaction, the dynamical systems approach operates at the systems level of explanation. From a traditional (e.g., cognitivist) perspective, thinking about how to change behavior at the individual level, for example, could be problematic from a dynamical systems perspective.

An example is the development of training programs that seek to alter a worker’s KSAs in order to improve performance and outcomes (Salas et al., 2006). In the standard approach, the KSAs to be trained should be understandable to both the trainer and the trainee. The reason for this is that we must be able to understand what we are doing incorrectly if we are to change our behavior, and we must be able to observe whether our behavior has actually changed. But if behavior is a function of real-time interactions and not just KSAs, how do we change it? Turning to dynamics, it seems difficult to identify a particular KSA that we can instruct individuals on to, say, alter the long-memory effects or power laws that inform their behavior. While we can observe changes in the dynamics, it could prove challenging to provide instructions to an individual about how their local behavioral variability contributes to and is constrained by global dynamics over long timescales.

Individual training is critical, but it is only realized in the context of real-time interpersonal dynamics between an individual and their teammates, where constructs such as KSAs must be understood in the context of the stable states of a team’s attractor dynamics. Formal equations of change in individual psychological states embedded in the interactions of dynamical systems have predicted individual variation in domains such as personality (Nowak et al., 2005) and marital satisfaction (Gottman et al., 2002), and similar equations have been written for teams (Guastello, 2017). As Nowak et al. (2005) point out, individual-level properties, such as KSAs, can give meaning to or modulate global dynamics, but more precisely, an individual’s behavior is variable in order to converge on stable states of the entire system (Principle 1).

Within the context of the dynamical systems approach, individual thought and behavior are a function of real-time team interactions, in which KSAs or other individual-level properties are embedded. Individual-level properties are considered “intrinsic dynamics” and are a part of the initial conditions of the system (Nowak et al., 2005), but the way that thought and behavior play out can only be realized in the context of real-time team interactions. Returning to the concept of “mechanism,” future research should not try to isolate dynamical principals in terms of reductionist psychological mechanisms such as working memory or pools of attentional resources. Rather, the notion of a psychological mechanism must continue to be extended to include dynamical principles that structure individual-level variability. Dynamical mechanisms (Peng et al., 1995) include attractor formation and dynamics, synchronization, and fractal scaling of thought and behavior. Future research should continue to study these “systems-level” psychological mechanisms through methods such as perturbation training and real-time team communication dynamics, as described above.

Separate from this, we think there are some interesting future directions that the dynamical systems approach entails from a cognitivist perspective. For example, investigating the questions of What do people actually know about the dynamics they produce, and Can they learn to control them? might enhance training at the level of individual-level properties. In terms of training, answering these questions could allow for the control of unintentional behaviors that interpersonal dynamics produce (e.g., spontaneous synchronization) and might provide individuals insight into the global, systems-level nature of their local behaviors (e.g., how their local behaviors are constrained by global coordination patterns). One might think of this as metacognition for systems or, perhaps, systems thinking from the perspective of an element within the system.

## Conclusion

In summary, it is important to recognize how interactions shape our thoughts and behaviors. It is critical to understand this because so much of what we do involves interacting with other people and technologies that automate what people do. Dr. Martin Luther King Jr once wrote, “We are caught in an inescapable network of mutuality…. Whatever affects one directly affects all indirectly” (King, 1963). Ultimately, we think that understanding how dynamic interaction processes shape our thoughts and behaviors is a fundamental psychological question that is at the heart of understanding human nature.

In this article we have presented dynamical systems concepts and how they can be used to understand and model teams. Our results thus far have converged on three principles underlying human performance in teams. We present them in abbreviated form here:

(1) Local variability ensures global stability and vice versa.
(2) These dynamics are substrate-independent; there is no preferred level of analysis.
(3) Cross-level effects occur between levels of analysis.

That global team patterns vary in predictable ways is not a proxy for individual KSAs that have to exist in order to perform a task, but it provides systems-level explanations for how real-time interaction processes shape thought and behavior. Where, then, does team behavior come from? Based on our research, we think that the ontology that interpersonal behavior and teamwork are somehow encoded in the individual is inaccurate; rather, what is encoded in the individual emerges out of a vibrant network or interpersonal, social, and cultural interactions that continuously shape and reshape that which is encoded (Bakhtin, 1986). From this perspective, not just teams but individuals in any interactive environment can be understood and modeled using a systems approach.

## Author Contributions

JG primarily wrote the paper. TD assisted with the cognitive-behavioral and cross-level effects sections. DG assisted with the real-time analysis section. CG assisted with the dynamical concepts and unintentional synchronization sections. All authors contributed to the conceptualization and outline of the paper.

## Conflict of Interest Statement

The authors declare that the research was conducted in the absence of any commercial or financial relationships that could be construed as a potential conflict of interest.

## References

[B1] Abney D. H. Paxton A. Dale R. Kello C. T. (2014). Complexity matching in dyadic conversation. *J. Exp. Psychol.* 143 2304–2315. 10.1037/xge01000021 25285431

[B2] Abraham R. Shaw C. D. (1992). *Dynamics: The Geometry of Behavior*, 2nd Edn. Boston, MA: Addison-Wesley.

[B3] Amazeen P. G. Schmidt R. C. Turvey M. T. (1995). Frequency detuning of the phase entrainment dynamics of visually coupled rhythmic movements. *Biol. Cybern.* 72 511–518. 10.1007/BF00199893 7612722

[B4] Bak P. (1996). *How Nature Works: The Science of Self-Organized Criticality*. New York, NY: Copernicus. 10.1007/978-1-4757-5426-1

[B5] Bakhtin M. M. (1986). *Speech Genres and other Late Essays* (eds Emerson C. Holquist M. McGee trans. V. W.). Austin, TX: University of Texas Press.

[B6] Barnard P. May J. Duke D. Duce D. (2000). Systems, interactions, and macrotheory. *ACM Trans. Comput. Hum. Interact.* 7 222–262. 10.1145/353485.353490

[B7] Beran J. (1994). *Statistics for Long-Memory Processes*, Vol. 61 New York, NY: Chapman & Hall

[B8] Bermas H. Fenoglio M. Haun W. Moore J. T. (2004). Laparoscopic suturing and knot tying: a comparison of standard techniques to a mechanical assist device. *JSLS* 8 187–189. 15119668 PMC3015531

[B9] Blickensderfer E. Cannon-Bowers J. A. Salas E. (1998). “Cross-training and team performance,” in *Making Decisions Under Stress: Implications for Individual and Team Training*, eds Cannon-Bowers J. A. Salas E. (Washington, DC: American Psychological Association), 299–311. 10.1037/10278-011

[B10] Bourbousson J. Sève C. McGarry T. (2010). Space-time coordination dynamics in basketball: part 1. Intra- and inter-couplings among player dyads. *J. Sports Sci.* 28 339–347. 10.1080/02640410903503632 20131146

[B11] Brown C. T. Liebovitch L. S. (2010). *Fractal Analysis.* Los Angeles, CA: SAGE 10.4135/9781412993876

[B12] Butner J. Pasupathi M. Vallejos V. (2008). When the facts just don’t add up: the fractal nature of conversational stories. *Soc. Cogn.* 26 670–699. 10.1521/soco.2008.26.6.670

[B13] Card S. Moran T. P. Newell A. (1983). *The Psychology of Human Computer Interaction.* Mahwah, NJ: Lawrence Erlbaum Associates.

[B14] Chapanis A. (1996). *Human Factors in Systems Engineering.* New York, NY: John Wiley & Sons, Inc.

[B15] Chartrand T. L. Bargh J. A. (1999). The chameleon effect: the perception-behavior link and social interaction. *J. Pers. Soc. Psychol.* 76 893–910. 10.1037/0022-3514.76.6.893 10402679

[B16] Coey C. A. Washburn A. Hassebrock J. Richardson M. (2016). Complexity matching effects in bimanual and interpersonal syncopated finger tapping. *Neurosci. Lett.* 616 204–210. 10.1016/j.neulet.2016.01.066 26840612 PMC4810785

[B17] Collins J. J. De Luca C. J. (1995). Upright, correlated random walks: a statistical-biomechanics approach to the human postural control system. *Chaos* 5 57–63. 10.1063/1.166086 12780156

[B18] Cooke N. J. Gorman J. C. (2009). Interaction-based measures of cognitive systems. *J. Cogn. Eng. Dec. Mak.* 3 27–46. 10.1518/155534309X433302

[B19] Cooke N. J. Gorman J. C. Myers C. W. Duran J. L. (2013). Interactive team cognition. *Cogn. Sci.* 37 255–285. 10.1111/cogs.12009 23167661

[B20] Cooke N. J. Gorman J. C. Rowe L. J. (2009). “An ecological perspective on team cognition,” in *Team Effectiveness in Complex Organizations: Cross-Disciplinary Perspectives and Approaches. SIOP Organizational Frontiers Series*, eds Salas E. Goodwin J. Burke C. S. (New York, NY: Taylor & Francis), 157–182.

[B21] Cuijpers L. S. Zaal F. T. de Poel H. J. (2015). Rowing crew coordination dynamics at increasing stroke rates. *PLoS ONE* 10:e0133527 10.1371/journal.pone.0133527 PMC4505883 26185987

[B22] DeChurch L. A. Mesmer-Magnus J. R. (2010). The cognitive underpinnings of effective teamwork: a meta-analysis. *J. Appl. Psychol.* 95 32–53. 10.1037/a0017328 20085405

[B23] Den Hartigh R. J. R. Gernigon C. Van Yperen N. W. Marin L. Van Geert P. L. C. (2014). How psychological and behavioral team states change during positive and negative momentum. *PLoS ONE* 9:e97887 10.1371/journal.pone.0097887 PMC4023954 24838238

[B24] Dunbar T. A. Gorman J. C. (2014). Fractal effects of task constraints in the self-organization of team communication. *Talk Presented at the Human Factors and Ergonomics Society 58th Annual Meeting*, Chicago, IL.

[B25] Fine J. M. Likens A. D. Amazeen E. L. Amazeen P. G. (2015). Emergent complexity matching in interpersonal coordination: local dynamics and global variability. *J. Exp. Psychol.* 41 723–737. 10.1037/xhp0000046 25798782

[B26] Frank T. D. Michelbrink M. Beckmann H. Schöllhorn W. I. (2007). A quantitative dynamical systems approach to differential learning: self-organization principle and order parameter equations. *Biol. Cybern.* 98 19–31. 10.1007/s00422-007-0193-x 18026746

[B27] Furuya S. Kinoshita H. (2008). Organization of the upper limb movement for piano key-depression differs between expert pianists and novice players. *Exp. Brain Res.* 185 581–593. 10.1007/s00221-007-1184-9 17989970

[B28] Furuya S. Soechting J. F. (2012). Speed invariance of independent control of finger movements in pianists. *J. Neurophysiol.* 108 2060–2068. 10.1152/jn.00378.2012 22815403 PMC3545004

[B29] Gibson J. J. (1966). *The Senses Considered as Perceptual Systems.* Boston, MA: Houghton Mifflin.

[B30] Gilden D. L. Thornton T. Mallon M. W. (1995). 1/f noise in human cognition. *Science* 267 1837–1839. 10.1126/science.7892611 7892611

[B31] Gipson C. L. Gorman J. C. Hessler E. R. (2016). Top-down (prior knowledge) and bottom-up (perceptual modality) influences on spontaneous interpersonal coordination. *Nonlinear Dynamics Psychol. Life Sci.* 20 193–222. 27033133

[B32] Gorman J. C. (2005). “The concept of long memory in assessing the global effects of augmented team cognition,” in *Proceedings of the 11th International Conference on Human-Computer Interaction*, Las Vegas, NV, 22–27.

[B33] Gorman J. C. (2014). Team coordination and dynamics: two central issues. *Curr. Dir. Psychol. Sci.* 23 355–360. 10.1177/0963721414545215

[B34] Gorman J. C. Amazeen P. G. Cooke N. J. (2010a). Team coordination dynamics. *Nonlinear Dynamics Psychol. Life Sci.* 14 265–289. 20587302

[B35] Gorman J. C. Amazeen P. G. Crites M. J. Gipson C. L. (2017). Deviations from mirroring in interpersonal multifrequency coordination when visual information is occluded. *Exp. Brain Res.* 235 1209–1221. 10.1007/s00221-017-4888-5 28188329

[B36] Gorman J. C. Cooke N. J. (2011). Changes in team cognition after a retention interval: the benefits of mixing it up. *J. Exp. Psychol.* 17 303–319. 10.1037/a0025149 21859230

[B37] Gorman J. C. Cooke N. J. Amazeen P. G. (2010b). Training adaptive teams. *Hum. Factors* 52 295–307. 10.1177/0018720810371689 20942257

[B38] Gorman J. C. Cooke N. J. Amazeen P. G. Fouse S. (2012a). Measuring patterns in team interaction sequences using a discrete recurrence approach. *Hum. Factors* 54 503–517. 10.1177/0018720811426140 22908675

[B39] Gorman J. C. Cooke N. J. Amazeen P. L. Hessler E. E. Rowe L. (2009). *Automatic Tagging of Macrocognitive Collaborative Processes through Communication Analysis.* Technical Report for Office of Naval Research Grant N00014-05-1–0625. Arlington, VA: Office of Naval Research.

[B40] Gorman J. C. Cooke N. J. Pederson H. K. Connor O. O. DeJoode J. A. (2005). Coordinated awareness of situation by teams (CAST): measuring team situation awareness of a communication glitch. *Proc. Hum. Fact. Ergon. Soc. Annu. Meet.* 49 274–277. 10.1177/154193120504900313

[B41] Gorman J. C. Cooke N. J. Winner J. L. (2006). Measuring team situation awareness in decentralized command and control environments. *Ergonomics* 49 1312–1325. 10.1080/00140130600612788 17008258

[B42] Gorman J. C. Crites M. J. (2015). Learning to tie well with others: bimanual versus intermanual performance of a highly practised skill. *Ergonomics* 58 680–697. 10.1080/00140139.2014.990523 25536870

[B43] Gorman J. C. Hessler E. E. Amazeen P. G. Cooke N. J. Shope S. M. (2012b). Dynamical analysis in real time: detecting perturbations to team communication. *Ergonomics* 55 825–839. 10.1080/00140139.2012.679317 22533819

[B44] Gorman J. C. Martin M. J. Dunbar T. A. Stevens R. H. Galloway T. L. Amazeen P. G. (2016). Cross-level effects between neurophysiology and communication during team training. *Hum. Factors* 58 181–199. 10.1177/0018720815602575 26391663

[B45] Gottman J. Swanson C. Swanson K. (2002). A general systems theory of marriage: nonlinear difference equation modeling of marital interaction. *Pers. Soc. Psychol. Rev.* 6 326–340. 10.1207/S15327957PSPR0604_07

[B46] Grehaigne J. F. Bouthier D. David B. (1997). Dynamic-system analysis of opponent relationships in collective actions in soccer. *J. Sports Sci.* 15 137–149. 10.1080/026404197367416 9258844

[B47] Grimm D. Gorman J. C. Stevens R. H. Galloway T. Willemsen-Dunlap A. M. Halpin D. J. (in press). “Demonstration of a method for real-time detection of anomalies in team communication,” in *Proceedings of the Human Factors and Ergonomics Society 61st Annual Meeting*, Austin, TX.

[B48] Guastello S. J. (2016). Physiological synchronization in a vigilance dual task. *Nonlinear Dynamics Psychol. Life Sci.* 20 49–80. 26639921

[B49] Guastello S. J. (2017). Nonlinear dynamical systems for theory and research in ergonomics. *Ergonomics* 60 167–193. 10.1080/00140139.2016.1162851 27097235

[B50] Guastello S. J. Marra D. E. Perna C. Castro J. Gomez M. Peressini A. F. (2016). Physiological synchronization in emergency response teams: subjective workload, drivers and empaths. *Nonlinear Dynamics Psychol. Life Sci.* 20 223–270. 27033134

[B51] Guru K. A. Sheikh M. R. Raza S. J. Stegemann A. P. Nyquist J. (2012). Novel knot tying technique for robot-assisted surgery. *Can. J. Urol.* 19 6401–6403. 22892267

[B52] Haken H. (1983). *Synergetics, an Introduction: Nonequilibrium Phase Transitions and Self-Organization in Physics.* New York, NY: Springer-Verlag. 10.1007/978-3-642-88338-5

[B53] Helmreich R. L. Merritt A. C. Wilhelm J. A. (1999). The evolution of Crew Resource Management training in commercial aviation. *Int. J. Aviat. Psychol.* 9 19–32. 10.1207/s15327108ijap0901_2 11541445

[B54] Hochberg J. (1986). “Representation of motion and space in video and cinematic displays,” in *Handbook of Perception and Human Performance*, eds Boff K. R. Kaufmann L. Thomas J. P. (New York, NY: Wiley), 22–21.

[B55] Hutchins E. (1996). *Cognition in the Wild.* Cambridge, MA: MIT Press.

[B56] Kantz H. Schreiber T. (2004). *Nonlinear Time Series Analysis*, 2nd Edn. Cambridge: Cambridge University Press. 10.1017/CBO9780511755798

[B57] Kean T. H. Hamilton L. H. (2004). *The 9/11 Commission Report.* Final Report of the National Commission on Terrorist Attacks Upon the United States. New York, NY: WW Norton and Company. 10.1002/j.1538-165X.2004.tb01293.x

[B58] Kelso J. A. S. (1995). *Dynamic Patterns: The Self-Organization of Brain and Behavior.* Cambridge, MA: MIT Press.

[B59] King M. L. Jr. (1963). *Letter from a Birmingham Jail.* Tuscaloosa, AL: University of Alabama.

[B60] Kozlowski S. W. J. Klein K. J. (2000). “A multilevel approach to theory and research in organizations: contextual, temporal, and emergent properties,” in *Multilevel Theory, Research, and Methods in Organizations: Foundations, Extensions, and New Directions*, eds Klein K. L. Kozlowski S. W. J. (San Francisco, CA: Jossey-Bass), 3–90.

[B61] Landauer T. K. Foltz P. W. Laham D. (1998). Introduction to latent semantic analysis. *Dis. Process.* 25 259–284. 10.1080/01638539809545028

[B62] Leonard H. B. Howitt A. M. (2006). Katrina as prelude: preparing for and responding to Katrina-class disturbances in the United States—Testimony to U.S. Senate Committee, March 8, 2006. *J. Homel. Secur. Emerg. Manag.* 3 1–20. 10.2202/1547-7355.1246

[B63] Leont’ev A. N. (1981). *Problems of the Development of Mind.* Moscow: Progress Publishers.

[B64] Liu Q. Kobayashi Y. Zhang B. Fujie M. G. (2014). “A novel smart surgical robotic system with eye-hand coordination for surgical assistance,” in *Proceeding of 2014 IEEE International Conference on Systems, Man, and Cybernetics (SMC2014)*, San Diego, CA, 1175–1180. 10.1109/SMC.2014.6974073

[B65] Mandelbrot B. B. (1967). How long is the coast of Britain? Statistical self-similarity and fractional dimension. *Science* 156 636–638. 10.1126/science.156.3775.636 17837158

[B66] Marks M. A. Sabella M. J. Burke C. S. Zaccaro S. J. (2002). The impact of cross-training on team effectiveness. *J. Appl. Psychol.* 87 3–13. 10.1037//0021-9010.87.1.3 11916213

[B67] Marmelat V. Deligniéres D. (2012). Strong anticipation: complexity matching in interpersonal coordination. *Exp. Brain Res.* 222 137–148. 10.1007/s00221-012-3202-9 22865163

[B68] McGrath J. E. Arrow H. Berdahl J. L. (2000). The study of groups: past, present, and future. *Pers. Soc. Psychol. Rev.* 4 95–105. 10.1207/S15327957PSPR0401_8

[B69] Morgan C. L. (2010). “Emergence,” in *Emergence, Complexity, and Self-Organization: Precursors and Prototypes*, eds Juarrero A. Rubino C. A. (Litchfield Park, AZ: Emergent Publications), 99–116.

[B70] Newell K. M. Liu Y.-T. Mayer-Kress G. (2001). Time scales in motor learning and development. *Psychol. Rev.* 108 57–82. 10.1037/0033-295X.108.1.57 11212633

[B71] Nowak A. Vallacher R. R. Zochowski M. (2005). The emergence of personality: dynamic foundations of individual variation. *Dev. Rev.* 25 351–385. 10.1016/j.dr.2005.10.004

[B72] Ouiller O. de Guzman G. C. Jantzen K. J. Lagarde J. Kelso J. A. S. (2008). Social coordination dynamics: measuring human bonding. *Soc. Neurosci.* 3 178–192. 10.1080/17470910701563392 18552971 PMC2156197

[B73] Peng C. K. Havlin S. Hausdorff J. M. Mietus J. E. Stanley H. E. Goldberger A. L. (1995). Fractal mechanisms and heart rate dynamics: long-range correlations and their breakdown with disease. *J. Electrocardiol.* 28 59–65. 10.1016/S0022-0736(95)80017-4 8656130

[B74] Peper C. E. Beek P. J. van Wieringen P. C. W. (1995). Multifrequency coordination in bimanual tapping: asymmetrical coupling and signs of supercriticality. *J. Exp. Psychol.* 21 1117–1138. 10.1037/0096-1523.21.5.1117

[B75] Quine W. V. O. (1951). Two dogmas of empiricism. *Philos. Rev.* 60 20–43. 10.2307/2181906

[B76] Ramachandran V. S. (2000). *Mirror Neurons and Imitation Learning as the Driving Force Behind “the Great Leap Forward” in Human Evolution.* Available at: http://www.edge.org/3rd_culture/ramachandran/ramachandran_index.html

[B77] Renshaw I. Chow J. Y. Davids K. Hammond J. (2010). A constraints-led perspective to understanding skill acquisition and game play: a basis for integration of motor learning theory and physical education praxis? *Phys. Educ. Sport Pedagogy* 15 117–137. 10.1080/17408980902791586

[B78] Richardson M. J. Marsh K. L. Isenhower R. W. Goodman J. R. L. Schmidt R. C. (2007). Rocking together: dynamics of intentional and unintentional interpersonal coordination. *Hum. Mov. Sci.* 26 867–891. 10.1016/j.humov.2007.07.002 17765345

[B79] Richardson M. J. Marsh K. L. Schmidt R. C. (2005). Effects of visual and verbal interaction on unintentional interpersonal coordination. *J. Exp. Psychol.* 31 62–79. 10.1037/0096-1523.31.1.62 15709863

[B80] Rizzolatti G. Fogassi L. Gallese V. (2001). Neurophysiological mechanisms underlying the understanding and imitation of action. *Nat. Rev. Neurosci.* 2 661–670. 10.1038/35090060 11533734

[B81] Rosenbaum D. A. (1998). Is dynamical systems modeling just curve fitting? *Motor Control* 2 101–104. 10.1123/mcj.2.2.101 9644279

[B82] Salas E. Dickinson T. L. Converse S. A. Tannenbaum S. I. (1992). “Toward an understanding of team performance and training,” in *Teams: Their Training and Performance*, eds Swezey R. W. Salas E. (Norwood, NJ: Ablex), 3–29.

[B83] Salas E. Wilson K. A. Priest H. A. Guthrie J. W. (2006). “Design, delivery, and evaluation of training systems,” in *Handbook of Human Factors and Ergonomics*, 3rd Edn, ed. Salvendy G. (Hoboken, NJ: John Wiley & Sons), 472–512. 10.1002/0470048204.ch18

[B84] Schmidt R. A. Bjork R. A. (1992). New conceptualizations of practice: common principles in three paradigms suggest new concepts for training. *Psychol. Sci.* 3 207–217. 10.1111/j.1467-9280.1992.tb00029.x

[B85] Schmidt R. C. Carello C. Turvey M. T. (1990). Phase transitions and critical fluctuations in the visual coordination of rhythmic movements between people. *J. Exp. Psychol.* 16 227–247. 10.1037/0096-1523.16.2.227 2142196

[B86] Schöllhorn W. I. Beckmann H. Michelbrink M. Sechelmann M. Trockel M. Davids K. (2006). Does noise provide a basis for the unification of motor learning theories? *Int. J. Sports Psychol.* 37 186–206.

[B87] Schroeder M. (2009). *Fractals, Chaos, Power Laws: Minutes from an Infinite Paradise.* Mineola, NY: Dover.

[B88] Shannon C. Weaver W. (1949). *The Mathematical Theory of Communication.* Urbana: University of Illinois Press. 10.1002/j.1538-7305.1948.tb01338.x

[B89] Shockley K. (2005). “Cross recurrence quantification of interpersonal postural activity,” in *Tutorials in Contemporary Nonlinear Methods for the Behavioral Sciences*, eds Riley M. A. Orden G. C. Van (Arlington, VA: Digital Publication Available through the National Science Foundation), 142–177.

[B90] Smith P. A. Baber C. Hunter J. Butler M. (2008). Measuring team skills in crime scene investigation: exploring ad hoc teams. *Ergonomics* 51 1463–1488. 10.1080/00140130802248076 18803089

[B91] Stephens G. J. Silbert L. J. Hasson U. (2010). Speaker-listener neural coupling underlies successful communication. *Proc. Natl. Acad. Sci. U.S.A.* 107 14425–14430. 10.1073/pnas.1008662107 20660768 PMC2922522

[B92] Stevens R. Galloway T. Gorman J. Willemsen-Dunlap A. Halpin D. (2016). “Toward objective measures of team dynamics during healthcare simulation training,” in *Proceedings of the International Symposium on Human Factors and Ergonomics in Health Care*, Orlando, FL. 10.1177/2327857916051010

[B93] Stevens R. H. Galloway T. (2014). Toward a quantitative description of the neurodynamic organizations of teams. *Soc. Neurosci.* 9 160–173. 10.1080/17470919.2014.883324 24502273

[B94] Stevens R. H. Galloway T. (2016). Modeling the neurodynamic organizations and interactions of teams. *Soc. Neurosci.* 11 123–139. 10.1080/17470919.2015.1056883 26079050

[B95] Stevens R. H. Galloway T. L. (2017). Are neurodynamic organizations a fundamental property of teamwork? *Front. Psychol.* 8:644 10.3389/fpsyg.2017.00644 PMC5411457 28512438

[B96] Strogatz S. H. (2004). *Sync: How Order Emerges from Chaos in the Universe, Nature, and Daily Life.* New York, NY: Hyperion.

[B97] Thelen E. Smith L. B. (1994). *A Dynamic Systems Approach to the Development of Cognition and Action.* Cambridge, MA: MIT Press.

[B98] Treffner P. J. Kelso J. A. S. (1999). Dynamic encounters: long memory during functional stabilization. *Ecol. Psychol.* 11 103–137. 10.1207/s15326969eco1102_1

[B99] Treffner P. J. Turvey M. T. (1993). Resonance constraints on rhythmic movement. *J. Exp. Psychol.* 19 1221–1237. 10.1037/0096-1523.19.6.1221

[B100] Turvey M. T. (2009). On the notion and implications of organism-environment system. *Ecol. Psychol.* 21 97–111. 10.1080/10407410902877041

[B101] Van Orden G. C. Holden J. G. Turvey M. T. (2003). Self-organization of cognitive performance. *J. Exp. Psychol.* 132 331–350. 10.1037/0096-3445.132.3.331 13678372

[B102] Varlet M. Richardson M. J. (2015). What would be Usain Bolt’s 100-meter sprint world record without Tyson Gay? *J. Exp. Psychol.* 41 36–41. 10.1037/a0038640 25559749

[B103] Vaughan D. (1996). *The Challenger Launch Decision: Risky Technology, Culture, and Deviance at NASA.* Chicago, IL: The University of Chicago Press.

[B104] Vilar L. Araújo D. Davids K. Button C. (2012). The role of ecological dynamics in analysing performance in team sports. *Sports Med.* 42 1–10. 10.2165/11596520 22149695

[B105] Wertsch J. V. (1991). “A sociocultural approach to socially shared cognition,” in *Perspectives on Socially Shared Cognition*, eds Resnick L. B. Levine J. M. Teaseley S. D. (Washington, DC: American Psychological Association), 85–100. 10.1037/10096-004

[B106] Wiltshire T. J. Butner J. E. Fiore S. M. (2017). Problem-solving phase transitions during team collaboration. *Cogn. Sci.* 10.1111/cogs.12482 [Epub ahead of print]. 28213928

[B107] Zheng B. Swanström L. Mackenzie C. L. (2007). A laboratory study on anticipatory movement in laparoscopic surgery: a behavioral indicator for team collaboration. *Surg. Endosc.* 21 935–940. 10.1007/s00464-006-9090-y 17180265

